# The effects of work–family conflict, work engagement, and job burnout on self-rated health of public health emergency responders in Jilin Province, China, in the context of the COVID-19

**DOI:** 10.3389/fpubh.2024.1469584

**Published:** 2024-10-09

**Authors:** Bingqin Hu, Guofeng Yang, Jingyu Ma, Yitong Chen, Peiyao Cui, Yifang Liang, Xin He, Jinghua Li

**Affiliations:** ^1^School of Public Health, Jilin University, Changchun, Jilin Province, China; ^2^Center for Disease Control and Prevention of Xizang Autonomous Region, Lhasa, Xizang Autonomous Region, China

**Keywords:** public health emergency responders, work–family conflict, work engagement, job burnout, self-rated health

## Abstract

**Introduction:**

Amid sudden public health crises, preserving the well-being and optimal working states of frontline healthcare professionals is imperative for efficaciously managing the emergences. However, there is a paucity of research investigating the health status of frontline healthcare professionals through the perspective of work–family conflict. This study sought to elucidate the complex interrelations between work–family conflict, work engagement, job burnout, and self-rated health among public health emergency responders within the context of the COVID-19 pandemic.

**Methods:**

A convenience sampling method was employed to survey 1,309 public health emergency responders at the Jilin Provincial Center for Disease Control and Prevention. An online survey was administered utilizing a self-constructed questionnaire. The hypothesized relationships between the variables were tested using structural equation modeling.

**Results:**

The direct impact of work–family conflict on self-rated health is not significant. The association between work–family conflicts and self-rated health was significantly mediated by work engagement and job burnout, respectively. Meanwhile, work engagement and job burnout had a chain mediating effect on work–family conflict and self-rated health.

**Conclusion:**

Work–family conflict plays a critical role in shaping the health and work status of public health emergency responders during public health crises. Organizations and managers should, in their workplace management practices, focus not only on work-related factors but also give due consideration to family-related factors. Supportive policies, including family-friendly initiatives, should be developed to safeguard the health and work engagement of public health emergency responders.

## 1 Introduction

The prolonged, global scale of the COVID-19 pandemic has introduced unparalleled challenges, severely disrupting the normal functioning of healthcare systems. As the healthcare system was impacted, the workload of frontline healthcare professionals surged rapidly within a compressed timeframe, while their working environment deviated significantly from typical conditions (1). They have faced work-related stressors and health risks that are markedly distinct from those encountered in earlier public health crises (2). Reports from multiple countries indicate that during the outbreak, frontline healthcare workers frequently endured excessive workloads, thereby increasing their risk of health complications (3, 4). Moreover, research evidence from multiple countries indicates that COVID-19 frontline workers exhibit pronounced symptoms of burnout, anxiety, and depression, thereby revealing a range of health issues (5–7). Consequently, safeguarding the operational capacity of healthcare professionals and strengthening the resilience of healthcare systems have become central concerns in emergency medical response. While the World Health Organization has declared that the COVID-19 outbreak is no longer considered a Public Health Emergency of International Concern, the pandemic has had enduring ripple effects on healthcare systems, disproportionately affecting frontline healthcare professionals. It is imperative for scholars to reflect on the pandemic and continue drawing insights from the experiences and lessons learned in order to better prepare for potential future crises.

Public health emergency responders constitute a crucial segment of the frontline workforce during sudden public health crises, playing an indispensable role in epidemic prevention and control, while safeguarding the overall health of the public (8). Especially in response to COVID-19, they undertook a comprehensive array of responsibilities, including conducting epidemiological surveys and data analysis, with the outcomes of their work directly influencing the formulation of epidemic prevention and control policies (9). In such a high-pressure environment, balancing rest, family responsibilities, and personal health becomes progressively challenging, ultimately resulting in emotional exhaustion, akin to that experienced by other frontline healthcare professionals. Investigating the relationships between work–family conflict, work engagement, and job burnout in relation to self-rated health, as well as the underlying mechanisms affecting public health emergency responders during the COVID-19 pandemic, is of profound importance. This research can yield valuable insights for policymakers in the post-pandemic era, providing critical guidance for strengthening future healthcare systems and optimizing emergency response frameworks. Furthermore, an extensive body of research has concentrated on work-related stress, job burnout, work engagement, and health issues among frontline workers (10–12). However, there is a dearth of research specifically examining the health issues impacting public health emergency responders from the perspective of work–family conflict. This article aims to explore how work–family conflict influences the self-reported health status of these responders from a novel perspective. Additionally, we aim to investigate how work engagement and job burnout mediate the relationship between work–family conflict and self-reported health status. Our study seeks to bridge gaps in current research and contribute to addressing policy limitations and enhancing care in this domain.

### 1.1 Work–family conflict and self-rated health

People often assume a variety of roles in life, of which work and family are two important domains. Work–family conflict (WFC) refers to the responsibilities of one domain creating stress and difficulties in the other domain (13), which consists of two dimensions: work-to-family conflict, family-to-work conflict. Work-family role conflicts have long been a part of our daily lives, with the expectations, obligations, and demands of different domains competing for individuals’ limited resources (14). Resource drain theory suggests that competition for a person’s limited resources can result in role conflict which in turn can have a variety of negative consequences (15, 16). Previous studies have indicated that work–family conflict can directly affect both physical and mental health, manifesting in symptoms like anxiety and depression (17, 18). Additionally, work–family conflict has several adverse effects, including heightened chronic stress and compromised immune function, the cumulative impact of which affects physical health (13, 19). Self-rated health is a comprehensive reflection of both physiological and psychological states, long regarded as a key indicator for evaluating individual health status, and extensively utilized in previous studies (20). Therefore, this study proposes the following hypotheses:

*Hypothesis* 1: Work–family conflict can negatively impact self-rated health.

### 1.2 Work engagement may mediate the relationship between work–family conflict and self-rated health

Work engagement is a positive work-related psychological sate, characterized by vigor, dedication, and absorption (21). As a positive emotional state, work engagement exhibits a significant correlation with various favorable work attitudes, behaviors, and health outcomes (22–24). According to conservation of resources theory, an individual’s work engagement depends on their personal resources, while work–family conflict may deplete an individual’s limited resources, making it challenging for them to maintain a high level of work engagement (25). Previous research suggests that demands from both work and family can create stress and have a negative impact on work engagement, indicating that work-family-related stress can affect individuals’ focus and reduce their level of work engagement (26). On the other hand, as a positive psychological trait, work engagement may have favorable effects on health (27). Research suggested that work engagement had beneficial effects on anxiety (28). Additionally, other study confirms a positive correlation between work engagement and self-rated health (29), with the mediating role of work engagement being validated. However, during the COVID-19 pandemic, public health emergency responders are faced with numerous work tasks, leaving them with little time and energy to fulfill their family responsibilities (30). This situation may lead to the occurrence of work–family conflict, which can adversely affect work engagement. As a result, the positive effects of work engagement may be inhibited, further negatively impacting their health. Therefore, this study proposes the following hypotheses:

*Hypothesis* 2: Work engagement serves as a mediator in the relationship between work–family conflict and self-rated health.

### 1.3 Job burnout may mediate the relationship between work–family conflict and self-rated health

Job burnout was a reaction to chronic interpersonal and emotional pressures at work (25). It is widely recognized that burnout is a slow, progressive loss of vigor and excitement (31). Due to the specialized nature of work in the public health emergency response sector, the completion of tasks often demands a combination of professional skills and emotional involvement (32). Based on conservation of resources theory (33), paying more working time and energy will decrease the participation of family, and will be accompanied by the decline in family support. Family and working domains will compete for individuals’ limited resources, then leads to the reduction of personal resources, finally the occurrence of job burnout (34). Previous studies have indicated that the impact of work–family conflict reduce individuals’ resources for coping with pressure, leading to the emergence of job burnout (35). Furthermore, the prolonged and persistent experience of job burnout has been validated to have negative implications for employees’ physical health (36). In the context of the COVID-19 pandemic, the heightened workload intensity has resulted in the increased job stress and work–family conflict. Concurrently, the decreased availability of family support and increased resource consumption have augmented the risk of job burnout, potentially affecting both their physical and mental well-being. Therefore, this study proposes the following hypotheses:

*Hypothesis* 3: Job burnout serves as a mediator in the relationship between work–family conflict and self-rated health.

### 1.4 Chain mediator of work engagement and job burnout

Work engagement, as a positive psychological state, has the capacity to counteract occupational burnout to some extent (37). Research has found that employees with higher levels of work engagement tend to associate the feelings of burnout with positive achievements, displaying a more enjoyable work state (38). The Job Demands-Resources Theory emphasizes the impact of job demands and job resources on an individual’s work-related outcomes (39). Adequate work resources can have a positive impact on individuals’ work, helping them engage more effectively, mobilize positive tendencies, and cope with stress and challenges (40). Conversely, high job demands can lead to physiological or psychological strain, resulting in fatigue and stress (41). Following the outbreak of COVID-19, the work environment of public health emergency responders experienced profound changes, particularly regarding workload and work pressure. They also encountered challenges such as resource and personnel shortages, along with diminished social support. In other words, the work demands for public health emergency responders have increased, while they were facing serious shortages in terms of work resources (42). Hence, public health emergency responders may encounter a decline in their level of work engagement owing to inadequate work resources and heightened job demands. The beneficial regulatory impact of work engagement could be constrained, potentially resulting in the onset of job burnout. Therefore, work engagement and job burnout might consecutively mediate the relationship between work–family conflict and self-rated health. Previous research has also confirmed the relationship between work engagement and occupational burnout. Based on this, this study proposes the following hypotheses:

*Hypothesis* 4: Work engagement and job burnout have a chain mediating effect in the relationship between work–family conflict and self-rated health.

## 2 Materials and methods

### 2.1 Procedure and study population

The study employed a cross-sectional survey design, conducted in Jilin Province, China, in 2021. Jilin, located in the high latitudes of northeastern China, and has a population exceeding 23.75 million. A questionnaire survey was administered to health emergency responders serving at the provincial, municipal, and county levels of the Centers for Disease Control and Prevention (CDCs), using a convenience sampling method. The study population included (1) public health emergency responders who were on duty at the time of the survey, and (2) full-time employees of the CDCs, excluding those who were employed on a temporary basis during the COVID-19 period. The questionnaire was distributed through an online survey platform^1^ with the assistance of the Jilin Provincial Health Care Commission. After obtaining informed consent from the participants, they completed the questionnaire either through WeChat or a dedicated webpage, ensuring anonymity and confidentiality. To ensure the quality of the data collected, the same IP can only answer once. A total of 1,314 participants took part in the study, of which 5 incomplete questionnaires were excluded, resulting in a validity rate of 99.6%. The study received approval from the Ethics Committee of the School of Public Health, Jilin University.

### 2.2 Measures

#### 2.2.1 Work–family conflict scale

The Work–Family Conflict Scale (WFCS) (43) was used to measure work–family conflict among health emergency responders. This scale assesses two dimensions: Work-to-family Conflict (WIF) and Family-to-work Conflict (FIW). Each of these dimensions contains three forms of conflict: time-based conflict, stress-based conflict, and behavior-based conflict. The WFCS consists of 18 items to be answered on a 5-point Likert scale, ranging from “strongly disagree” (1 point) to “strongly agree” (5 points). Higher scores indicate higher levels of work–family conflict. The Cronbach’s *α* coefficient for the WFCS in this study was 0.942.

#### 2.2.2 Work engagement scale

Work engagement was assessed using the Utrecht Work Engagement Scale (UWES), which consists of 17 items developed by Schaufeli (21). The UWES measures three dimensions: vigor, dedication, and absorption. Vigor was assessed through items such as “At my job, I feel strong and vigorous.” An example of dedication was “My job inspires me” and a sample item for absorption was “I am immersed in my work.” All items were answered on a 7-point Likert scale, ranging from “never” (0 point) to “every day” (6 points), with higher scores indicating higher levels of work engagement. The Cronbach’s *α* coefficient for the UWES in this study was 0.972.

#### 2.2.3 Job burnout scale

Job burnout was assessed using the Chinese version of the 15-item scale developed by Maslach et al. (MBI-GS) (31). The Chinese version of MBI-GS has been verified to have excellent reliability and validity (44, 45). The scale was divided into three dimensions: emotional exhaustion, depersonalization, and reduced personal accomplishment. Examples for each dimension include: “Work makes me feel like I’m going to collapse,” “Work makes me feel like I’m going to collapse,” and “I am confident that I can perform all tasks effectively.” All items were scored on a 7-point Likert scale, where “never” was scored as 0 and “always” was scored as 6 points. All items in the reduced personal accomplishment dimension were scored inversely. Higher scores indicated higher levels of burnout among health emergency responders. The Cronbach’s *α* coefficient for MBI-GS in this study was 0.907.

#### 2.2.4 Self-rated health

The self-rated health status was assessed using a single-item survey method, which has been widely employed in epidemiology and health services research (46). Its consistency with objective health status has been demonstrated in previous studies (47). In this study, participants self-rated health status was evaluated by asking them, “In general, how do you feel about your health?” They responded on a five-point Likert scale, which included options such as “poor,” “fair,” “good,” “very good” and “excellent” scored from 1 to 5. A higher score indicated a higher level of self-rated health.

#### 2.2.5 Covariates

The questionnaire included possible confounding variables that may be used as control variables when analyzing the association between work–family conflict and self-rated health. The variables mainly included gender (female or male), marital status (married or non-married), age subgroups (≤35 years; 36–45 years; ≥46 years), technical title (junior title and below (including no title); intermediate title; vice-senior or senior title), and whether had been involved in responding to emergency public health incidents. There was a correlation between gender, marital status, and the main variables. Therefore, gender and marital status were used as control variables in this study.

### 2.3 Statistical analysis

The data were analyzed using IBM SPSS version 26.0 and AMOS version 23.0. Descriptive analysis of participants’ demographics was conducted using IBM SPSS 26.0. Mean ± standard deviation (SD) was used to describe the scores on different scales. Independent sample *t*-tests and one-way analysis of variance (ANOVA) were employed to explore score differences among different categorical variables. A common method bias test was conducted utilizing Harman’s single-factor test and validation of the measurement models through confirmatory factor analysis (CFA). Additionally, Pearson correlation analysis was used to examine the relationships among the core variables in this study. Finally, to further investigate the hypothesized relationships among study variables, Structural Equation Modeling (SEM) was employed to analyze the associations between work–family conflict, work engagement, occupational burnout, and self-rated health. To assess the significance of the hypothesized model, a bootstrapping procedure with 2000 samples was utilized. When the 95% confidence interval did not include zero, the effect was considered significant. Model fit was evaluated using indices such as *χ^2^/df*, GFI, and CFI. The statistical significance level was set at *p* < 0.05.

## 3 Results

### 3.1 Sociodemographic characteristics

Table 1 presents both participant characteristics and the mean, standard deviation, and univariate analysis results for work–family conflict, work engagement, job burnout, and self-rated health. Most of the participants were women (64.7%). 78.0% of participants were married. The average age of the participants was 40.0 (SD = 10.12) years. The majority of participants held primary titles or below, accounting for 54.2% of the total. 74.2% of the participants indicated that they had participated in responding to emergency public health incidents. There are significant gender differences in work–family conflict scores. Work–family conflict, job burnout, and self-rated health scores exhibit significant variations among different age. Furthermore, self-rated health status scores show significant differences across various marital statuses. There are significant differences in self-rated health among participants with different technical titles. Lastly, work engagement scores significantly differ based on past participation in emergency public health events.

**Table 1:** Sociodemographic characteristics of the participants and univariate analysis for the research variables.

Variables	Total (*n* = 1,309)		Work–family conflict		Work engagement		Job burnout		Self-rated health	
	n	%	M ± SD	*p*	M ± SD	*p*	M ± SD	*p*	M ± SD	*p*
Gender										
Male	462	35.3	52.32 ± 15.59	0.000^***^	77.76 ± 21.79	0.419	27.71 ± 16.73	0.103	2.74 ± 1.18	0.059
Female	847	64.7	48.65 ± 13.89		76.75 ± 21.50		26.15 ± 16.27		2.61 ± 1.15	
Marital status										
Married	1,021	78.0	50.23 ± 14.13	0.205	77.01 ± 21.48	0.758	26.41 ± 16.23	0.235	2.59 ± 1.15	0.000^***^
Non-married	288	22.0	48.90 ± 16.19		77.45 ± 22.05		27.75 ± 17.16		2.92 ± 1.20	
Age										
≤35 yrs	504	38.5	50.72 ± 14.05	0.025^*^	77.65 ± 21.62	0.420	27.93 ± 17.22	0.001^*^	2.81 ± 1.19	0.001^*^
36-45 yrs	332	25.4	50.26 ± 12.59		75.77 ± 22.65		28.02 ± 16.13		2.52 ± 1.18	
≥46 yrs	473	36.1	48.43 ± 14.41		77.48 ± 20.82		24.47 ± 15.59		2.60 ± 1.12	
Technical titles				0.064		0.412		0.071		0.000^***^
Primary title or below	710	54.2	50.58 ± 16.06		77.84 ± 21.92		27.33 ± 17.25		2.83 ± 1.20	
Intermediate title	265	20.2	50.23 ± 12.54		76.14 ± 21.68		27.28 ± 15.65		2.43 ± 1.08	
Vice-senior or senior title	334	25.5	48.33 ± 12.70		76.33 ± 20.85		24.92 ± 15.17		2.49 ± 1.10	
Have you been involved in responding to emergency public health incidents										
Yes	971	74.2	50.40 ± 14.72	0.052	77.94 ± 21.12	0.018^*^	26.45 ± 16.43	0.338	2.63 ± 1.17	0.075
No	338	25.8	48.61 ± 14.22		74.71 ± 22.69		27.44 ± 16.50		2.76 ± 1.16	

M, mean; SD, standard deviation. *p < 0.05; ^**^p < 0.01; ^***^p < 0.001.

### 3.2 Confirmatory factor analysis and common method bias test

The results of the confirmatory factor analysis revealed that standardized factor loadings for each dimension exceeded 0.6, with composite reliability (C.R.) surpassing 0.9, and average variance extracted (AVE) values above 0.5. All the results mentioned above conformed to the validation standards set forth by Hair et al. and Fornell et al. (48, 49): ① factor loadings exceeding 0.5; ② composite reliability (C.R.) greater than 0.8; ③ AVE values exceeding 0.5. Harman’s single-factor test revealed that the first unrotated factor explained merely 33.25% of the total variance, falling below the established threshold of 40%, thereby indicating that the bias is maintained within an acceptable range.

### 3.3 Correlation analysis

The correlation coefficients between the variables are shown in Table 2. Work–family conflict was negatively related to job satisfaction and self-rated health, while it was positively related to burnout. The relationship between work engagement and burnout was negatively related, while it was positively related to self-rated health. Job burnout is negatively correlated with self-rated health.

**Table 2:** Scores on the scale and correlation coefficients among the variables of study.

Variable	M ± SD	Range	1	2	3	4
1.Work–family conflicts	49.94 ± 14.61	18–90	1			
2.Work engagement	77.11 ± 21.60	0–102	−0.134**	1		
3. Job burnout	26.70 ± 16.44	0–90	0.505**	−0.545**	1	
4. Self-rated health	2.66 ± 1.17	1–5	−0.113**	0.342**	−0.279**	1

M, mean; SD, standard deviation; **p < 0.01.

### 3.4 Mediation analyses

**Figure 1:**
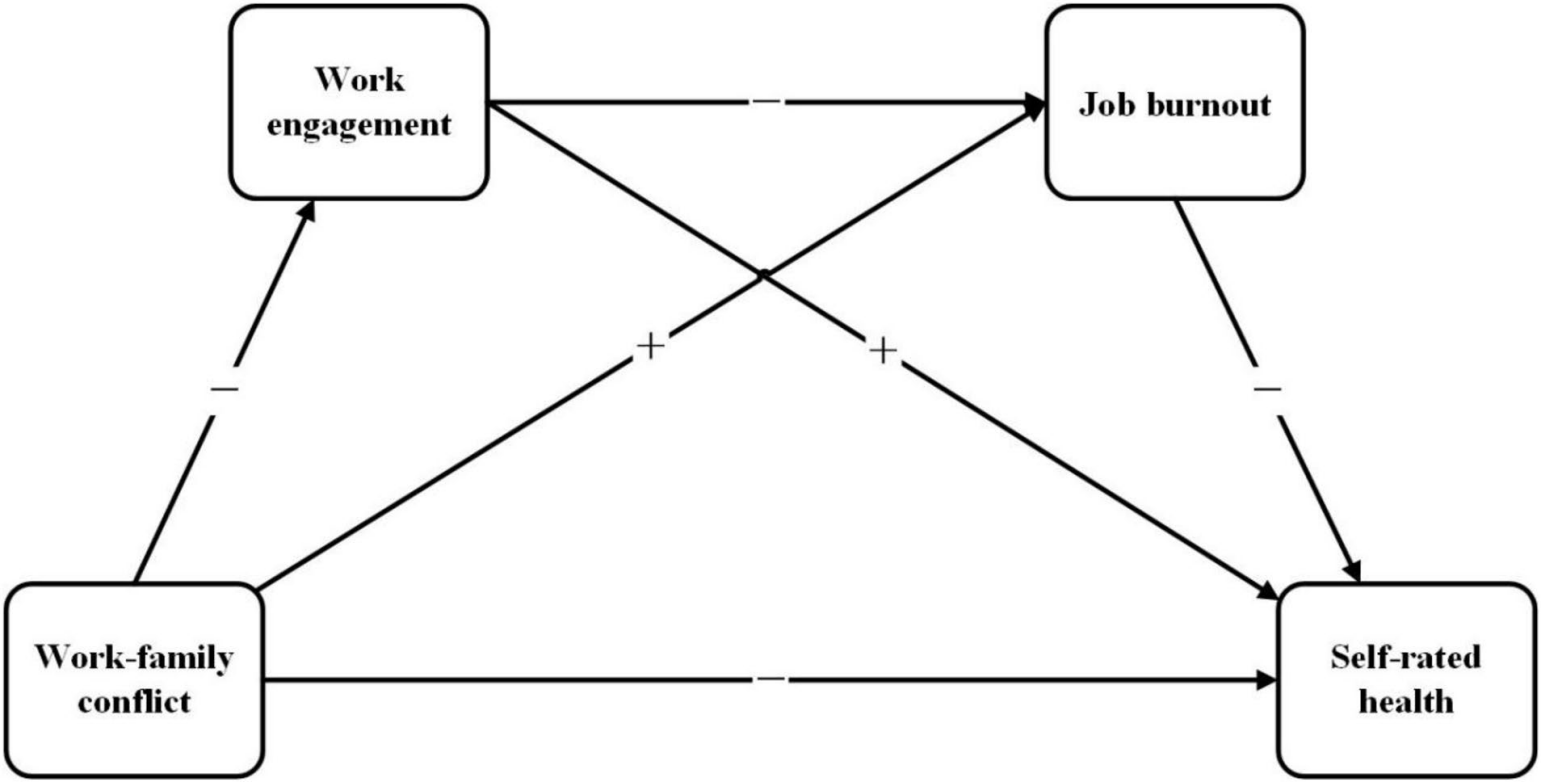
Hypothesis model.

**Figure 2:**
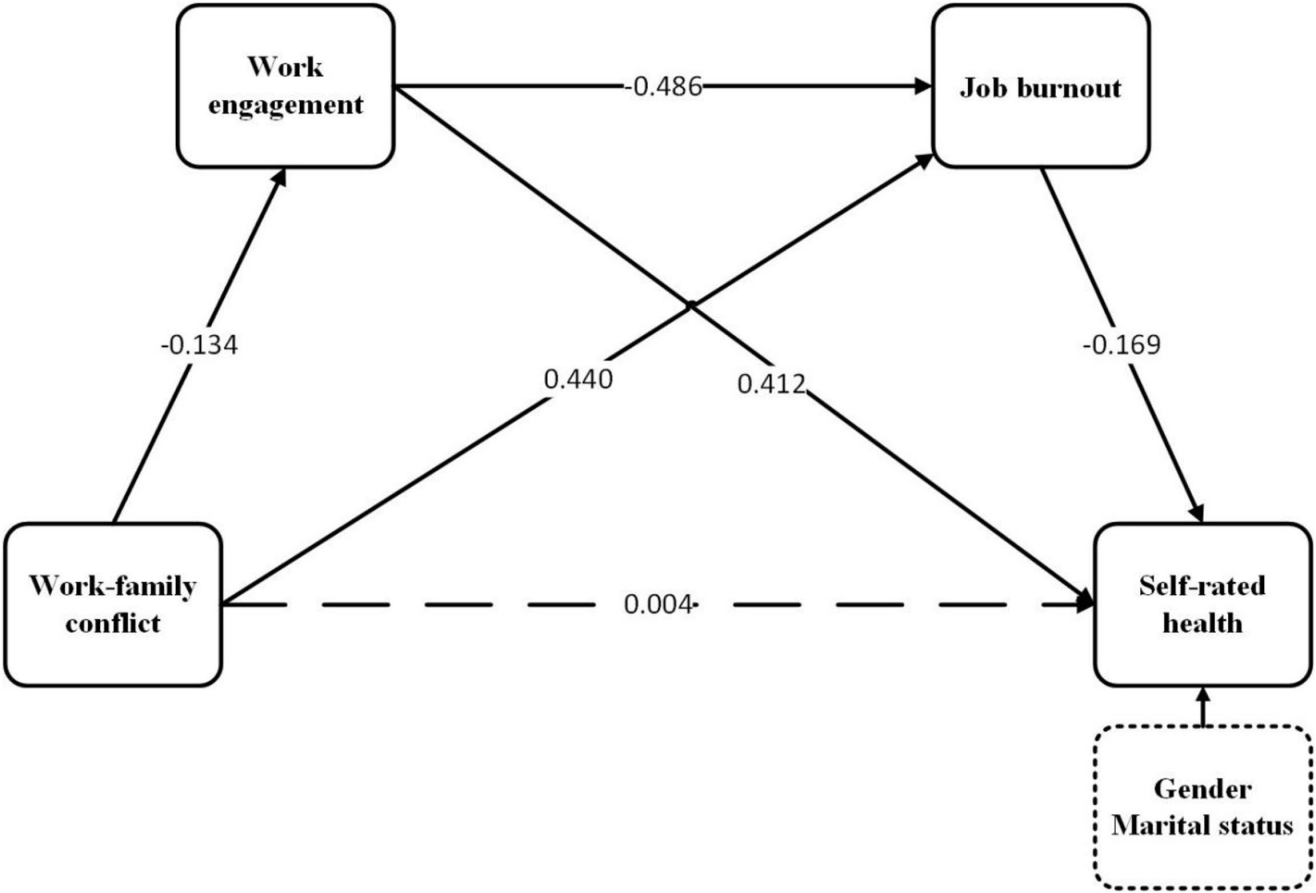
Chained mediation of work engagement and job burnout in the relationship between work–family conflict and self-rated health.

After controlling for two demographic sociological factors, gender and marital status, the final path is shown in Figure 1. According to the results, the model fit is good (χ^2^/*df* = 2.464, GFI = 0.998, CFI = 0.996, IFI = 0.996, TLI = 0.984, RMSEA = 0.033). The SEM analysis results for direct effects between work–family conflict, work engagement, job burnout, self-rated health were shown in Figure 2. The results showed that work–family conflict displayed a negative association with work engagement (*β* = −0.134, *p* < 0.001), while work–family conflict had a significant positive association with job burnout (*β* = 0.440, *p* < 0.001). Work engagement and job burnout had a significant negative effect on self-rated health (*β* = −0.412, *p* < 0.001; *β* = −0.169, *p* < 0.001), respectively. And work engagement had a significant negative effect on job burnout (*β* = 0.486, *p* < 0.001). Conversely, the direct effect of work–family conflict on self-rated health was not statistically significant.

Table 3 presents estimates for the total effect, direct, indirect along with bias-corrected 95% confidence interval. In terms of total effect, self-rated health exhibited a significant negative correlation with work–family conflict (*β* = −0.137, 95% CI [−0.208, −0.066]). Additionally, the bootstrap 95% confidence interval confirms the substantial indirect impact of work engagement and job burnout on the association between work–family conflict and self-rated health. These findings suggest that work engagement and job burnout not only partially mediate the link between work–family conflict and self-rated health but also exert a chain mediating effect on this relationship. However, contrary to the hypothesis, the direct effect of work–family conflict on self-rated health is not significant.

**Table 3:** The results of chained mediation analysis.

Effect	Pathways	Estimated effect	Boot SE	Boot LLCI	Boot ULCI
Total effect	Work–family conflict → Self-rated health	−0.137	0.037	−0.208	−0.066
Direct effect	Work–family conflict → Self-rated health	0.004	0.034	−0.067	0.071
Indirect effect	Work–family conflict → Work engagement →Self-rated health	−0.055	0.014	−0.082	−0.030
	Work–family conflict → Job burnout → Self-rated health	−0.074	0.017	−0.110	−0.042
	Work–family conflict →Work engagement → Job burnout → Self-rated health	−0.011	0.004	−0.020	−0.005

## 4 Discussion

This study aimed to investigate the impact of work–family conflict and its underlying mechanisms on self-rated health among public health emergency responders during the COVID-19 pandemic. Although the direct effect between work–family conflict and self-rated health was not significant, the mediating roles of work engagement and job burnout between work–family conflict and self-rated health were validated. Additionally, the chained mediation effect of work engagement and job burnout on the relationship between work–family conflict and self-rated health has been validated. This study elucidates the underlying mechanisms affecting public health responders’ self-perceived health related to work–family conflict, work engagement, and job burnout, thereby establishing a foundation for targeted interventions aimed at enhancing work engagement and mitigating occupational burnout. Furthermore, the findings yield critical insights and provide new evidence to bolster occupational protection measures for other frontline workers.

The average score for work–family conflict among public health emergency personnel is 49.94 ± 14.61, exceeding half of the total score and surpassing the score of a survey on medical personnel during non-sudden public health events (50). The results indicate that the conflict between work and family is a significant factor in the context of sudden public health events. In addition, the univariate results indicate significant differences in work–family conflict among public health emergency responders with varying sociodemographic characteristics. Gender differences in work–family conflict were observed, with men scoring higher than women in this domain. This phenomenon may be attributed to traditional gender roles and societal expectations that place greater work responsibilities on men (51). Consequently, men often encounter higher work demands and extended working hours, which can exacerbate work–family conflict (52). Additionally, differences were observed across various age groups, with respondents aged 35 and below reporting the highest levels of work–family conflict. This trend may be attributed to the relative lack of work experience among younger health emergency responders which renders it challenging for them to effectively balance work and family responsibilities (53). Moreover, this age group is within the childbearing years, which may lead to increased family responsibilities and demands, potentially exacerbating work–family conflict (54). Our research indicates that the sources of stress for frontline workers arise not only from their professional responsibilities but also from work–family conflict. This is particularly pronounced among young men, whose dual roles as breadwinners at work and at home may place them in an even more challenging position. Therefore, enhanced attention and support should be directed toward individuals experiencing elevated work or family stress, particularly young individuals of childbearing age and men in frontline roles. Such support may encompass family caregiving, early childhood care, and the provision of financial or living subsidies, among other measures.

This study suggests that in the relationship between work–family conflict and self-rated health, work engagement may serve as a crucial mediating factor. Work–family conflict can adversely impact work engagement, thereby diminishing the positive influence of work engagement on self-rated health status. Consistent with previous research findings (18, 28), work–family conflict can adversely affect work engagement, and the findings also corroborate the conservation of resources theory as well as role conflict theory. Both work and family require investment of time, energy, and other resources (2). In the context of the COVID-19 pandemic, work demands and task loads have markedly increased. The heightened consumption of resources in the work domain has further exacerbated the resource shortage in the family domain. This scarcity of resources makes it easier for conflicts between work and family to occur, thereby adversely affecting work engagement. Furthermore, the intensification of work–family conflict not only reduces work engagement but also adversely impacts self-rated health. This finding aligns with the fundamental tenet of role conflict theory, which posits that when the demands of one role conflict with those of another, it can lead to harmful consequences for an individual’s psychological and physical well-being. Therefore, increased attention and support for the families of frontline workers are essential to alleviate work–family conflict. Such policies will enhance the work engagement of frontline personnel and yield positive effects on their personal health. Good health not only reflects the safeguarding of their health rights but is also a prerequisite for effective public health emergency response (9, 55).

This study also found that work–family conflict had an indirect effect on self-rated health through job burnout. Job burnout is recognized as a significant indicator of employee turnover (50). Amid public health emergencies such as COVID-19, maintaining distinct boundaries between work and family becomes increasingly arduous, especially during prolonged periods of high-intensity work. This ongoing strain depletes individual resources, leading to job burnout and adversely affecting health status (56). Furthermore, various types of contradictions contribute to the emotional exhaustion of frontline personnel, potentially leading to turnover intentions or actual turnover, thereby destabilizing the healthcare system. Our research confirms the impact of family factors on individual job burnout and health status, a consideration frequently overlooked in prior studies. Additionally, it suggests that family-friendly policies can serve as effective interventions to mitigate job burnout, enhance health status, and reduce turnover, particularly during health emergencies. Concerted efforts should be undertaken to optimize the work schedules of public health emergency personnel by alleviating excessively long working hours and overwhelming workloads through the allocation of adequate human resources, thereby ensuring ample time for rest and recovery. Moreover, it is crucial to regularly train and maintain a sufficient pool of healthcare professionals to guarantee an adequate supply of personnel in response to crises. Finally, in the absence of sufficient family support, it is imperative to provide adequate psychological assistance, including enhanced emergency psychological services, and regular monitoring and assessment of their mental health status.

The chained mediation effect of work engagement and job burnout between work–family conflict and self-rated health has been validated. Consistent with our findings, previous studies have demonstrated that work engagement negatively impacts job burnout (57). Bereznowski et al. found that work engagement can also alleviate job burnout (58). Individuals with high levels of work engagement are more likely to maintain a positive work attitude, thereby reducing job burnout. This reduction in burnout can subsequently have beneficial effects on their health, fostering a virtuous cycle (59). Work–family conflict marks the beginning of a virtuous circle; therefore, it is crucial to reduce its incidence. It is important to provide public health emergency responders with sufficient resources and support, at both the individual and family levels, to ensure their well-being and good health. Furthermore, fostering a supportive work environment is essential, particularly in high-pressure contexts. Establishing open channels of communication that enable employees to articulate their needs and challenges can significantly mitigate work–family conflict. A stable, healthy, and sufficient health emergency response workforce is crucial, not only in non-emergency situations through stockpiling and training but also in emergencies by providing them with material and moral resources to minimize depletion, thereby reducing the occurrence of work–family conflicts.

## 5 Limitations

This research had some limitations. Firstly, we used a cross-sectional study due to the epidemic and time constraints, which was insufficient to explain the causal relationship between the study variables. Second, we conduct surveys using web-based questionnaires rather than face-to-face surveys, and it is difficult to ensure consistency with the actual situation using self-administered web-based questionnaires. Third, the study’s sample is from the same province, and the findings may not be applicable to other areas due to differences in epidemic prevention and control pressures and policies across provinces. Fourth, the scope of our study sample was restricted solely to public health emergency responders. Future investigations could consider broadening the scope to encompass a wider range of occupational groups, including but not limited to physicians, nurses, and other professionals.

## 6 Conclusion

This current study investigated how work–family conflicts, work engagement and job burnout affect self-rated health among health emergency workers. The direct effect of work–family conflicts on self-rated health was not significant. Work–family conflicts indirectly affects public health emergency responders’ self-rated health through the mediating effect of work engagement and job burnout. The findings contribute to a better understanding of the pathways of action of how work–family conflict affects the self-rated health status of public health emergency responders. To protect the health of public health emergency responders, effective interventions to reduce work–family conflict can be implemented.

## Data Availability

The raw data supporting the conclusions of this article will be made available by the authors, without undue reservation.

## Footnotes

^1^ https://www.wjx.cn/
